# Application of Surfactant Modified Natural Zeolites for the Removal of Salicylic Acid—A Contaminant of Emerging Concern

**DOI:** 10.3390/ma14247728

**Published:** 2021-12-14

**Authors:** Danijela Smiljanić, Aleksandra Daković, Milena Obradović, Milica Ožegović, Francesco Izzo, Chiara Germinario, Bruno de Gennaro

**Affiliations:** 1 Institute for Technology of Nuclear and Other Mineral Raw Materials, Franche D’ Epere 86, 11000 Belgrade, Serbia; a.dakovic@itnms.ac.rs (A.D.); m.obradovic@itnms.ac.rs (M.O.); m.spasojevic@itnms.ac.rs (M.O.); 2 Department of Earth Sciences, Environment and Resources, Federico II University, Via Cinthia, 80126 Napoli, Italy; francesco.izzo4@unina.it; 3 Department of Science and Technology, University of Sannio, Via F. De Sanctis, 82100 Benevento, Italy; chiara.germinario@unisannio.it; 4 Department of Chemical, Materials and Production Engineering, Federico II University, Piazzale V. Tecchio 80, 80125 Naples, Italy; bruno.degennaro@unina.it

**Keywords:** surfactant modified natural zeolites (SMNZs), emerging contaminants, salicylic acid

## Abstract

This work aimed to test composites (surfactant modified zeolites prepared by treatment of natural zeolites—clinoptilolite (IZ CLI) and/or phillipsite (PHIL75)-rich tuffs with two different amounts of cationic surfactants: cetylpyridinium chloride (CPyCl) and Arquad^®^ 2HT-75 (ARQ)) for the adsorption of salicylic acid (SA)—a common contaminant of emerging concern. Adsorption of SA was studied at different initial drug concentrations (in the range of 2–100 mg/L) in water solution. The Langmuir isotherm model showed the highest adsorption was achieved by bilayer composite of IZ CLI and CPyCl—around 11 mg/g. Kinetic runs were performed by using the initial drug concentration of 20 mg/L in the time interval from 0 to 75 min and pseudo-second order had good correlation with experimental data. The influence of the four different temperatures on the SA adsorption was also investigated and thermodynamic parameters suggested that the adsorption drug onto composites is an exothermic and nonspontaneous process, followed by the decrease of randomness at the solid/liquid interface during the adsorption. Zeta potential and Fourier-transform infrared spectroscopy with attenuated total reflectance (FTIR-ATR) had been performed for the characterization of composites after adsorption of SA confirming the presence of the drug at composite surfaces.

## 1. Introduction

Emerging contaminants (ECs) are a large group of naturally occurring or manmade compounds present in the environment whose toxicity or persistence could affect the health of living beings [1]. Several groups are considered as ECs: pharmaceuticals, steroid hormones, personal care products, pesticides, surfactants, fragrances, plasticizers, flame retardants, nanoparticles, perfluoroalkyl compounds, algal toxins, various trace elements such as rare earths, and radionuclides [2,3,4,5]. Currently, these compounds are not subjected to any discharge limitations; however, ECs are becoming a growing concern due to their often detection in different water bodies in concentrations from a few ng/L to a few hundred µg/L, and even mg/L in industry effluents [3,4,6]. Effluents from wastewater treatment plants (WWTPs) are a significant source of ECs since conventional treatment is often inadequate for removal of these pollutants [7,8,9], this enables ECs to further spread through surface water and ultimately to reach drinking water, potentially affecting human health [4,10]. Different techniques have been used for water treatment such as membrane filtration, advanced oxidation processes (oxidation, ozonation, ultrasonic irradiation, etc.), electrochemical and biological processes, etc. [11,12]. However, each method has some disadvantages, like high operational costs, formation of by-products, or complexity of the process [11,12,13,14]. Therefore, adsorption—as a low-cost, simple, environmentally friendly, and by-product free option—is a promising technique [12,14,15].

Natural zeolites are extensively studied as adsorbents of heavy metal cations [16,17,18,19,20]. However, these minerals have no affinity toward low-polar molecules and anions; thus, to overpower this limitation zeolitic surface can be modified with cationic surfactants producing surfactant modified natural zeolites (SMNZs). At the zeolitic surface, molecules of cationic surfactant can be arranged in monolayer or bilayer, depending on the surfactant amount used for modification as well as the counter ion effects [21,22]. Monolayer composites (containing surfactants in amounts up to 100% of ECEC) have an affinity towards hydrophobic molecules, whereas bilayers (with amounts of surfactant above ECEC value) besides hydrophobic molecules could also adsorb anions [23,24,25,26]. Consequently, SMNZs have the ability to simultaneously adsorb different pollutants: cations, anions, organic molecules, and pathogens [15,20,27,28,29,30,31].

Among the pharmaceuticals, especially important are antibiotics and steroid hormones, but also nonsteroidal anti-inflammatory drugs (NSAIDs): diclofenac (DCF), ibuprofen (IBU), salicylic acid (SA), naproxen (NAP), etc. These compounds are frequently detected in water because of their massive consumption [3,4,32,33]. SMNZs have been examined for pharmaceutical purposes—as carriers of NSAIDs [21,34,35,36,37,38,39]. Some of the findings were that amounts of adsorbed DCF and IBU were around a few dozen of mg/g and that this number increased when the initial drugs concentrations increased [21,34,35,36,37,38,39]. However, literature about the application of SMNZs for the removal of NSAIDs from water is sparse [40,41,42]. In our previous studies, removal of DCF, KET, IBU, and NAP using SMNZs prepared by modification of clinoptilolite and phillipsite-rich tuffs with cetylpyridinium chloride (CPyCl) and Arquad^®^ 2HT-75 (ARQ) (100% and 200% of ECEC values) were reported [41,42]. Characterization of obtained composites by Fourier-transform infrared spectroscopy with attenuated total reflectance (FTIR–ATR), simultaneous thermal analysis (STA) with evolved gases analysis (EGA), and determination of zeta potential, confirmed the presence of surfactants at clinoptilolite and phillipsite surfaces. It was pointed that DCF, KET, IBU, and NAP adsorption increased with increasing the amount of either CPyCl or ARQ in composites. The highest amounts of drugs were achieved with the composites with the highest level of surfactants. Results confirmed the effectiveness of adsorbents for the removal of the four mentioned drugs.

Salicylic acid, metabolite of acetylsalicylic acid (ASA)—the active component of aspirin (in vivo hydrolyzed to SA)—is used in cosmetic products due to its keratolytic and antifungal properties [3,7,12,43,44,45]. The use of SA in high concentrations can cause severe health problems and even death (3.1 deaths per 1000 aspirin ingestions compared to 0.1 per 1000 IBU ingestions) [12,44]. For this reason, the presence of SA in water, frequently detected in concentrations from ng/L to µg/L depending on water type (WWTPs influent/effluents, hospital waste, pharmaceutical industry effluents, surface water, etc.) is a rising concern, and its removal from water is of great importance [5,7,12,32,43,46,47,48,49]. Between three metabolites of ASA, SA had intermediate acute toxicity to daphnids (EC_50_ values were 1945.32 and 1147.57 mg/L for *Daphnia magna* and *Daphnia longispina*, respectively), whereas chronic exposures to SA affected normal reproduction or growth in both types of cladocerans [43]. Another study has shown no acute toxicity for species *Daphnia magna* and bacterium *Vibrio fisheri*, at two tested concentrations of 32 µg/L and 500 µg/L as well as no chronic toxicity (tested on *Ceriodaphnia dubia* and *Selanastrum capricornutum* at concentration 32 µg/L) [48]. Literature data on application of SMNZs as adsorbents for SA are rare. Only, clinoptilolite modified with benzalkonium chloride (in amount 500% of ECEC value) was tested as carrier of SA [50], while a commercial zeolite modified with hexadecyltrimethylammonium chloride—HDTMA (100% of ECEC value) was investigated as adsorbent of SA [50,51].

The present study aims to examine if the previously prepared composites of clinoptilolite and phillipsite-rich tuffs with CPyCl and ARQ (100% and 200% of ECEC values) are capable to adsorb SA, a metabolite of a common anti-inflammatory drug ASA. The influence of structure of surfactants (CPyCl has aromatic ring and single alkyl chain, while ARQ has two alkyl chains) on adsorption of SA was evaluated. The drug adsorption experiments were performed in distilled water, under the following conditions: different initial drug concentration, contact time, and temperature. The prepared composites were used for the first time for SA adsorption. The mechanism of SA adsorption by composites was also suggested based on the drug adsorption experiments as well as on the characterization of composites after SA adsorption.

## 2. Materials and Methods

Natural zeolite from Turkey (IZ CLI) with 79 wt % of clinoptilolite and a zeolitic-rich tuff from Italy (PHIL75), with 58 wt % of zeolite content (mainly ca. 44 wt % of phillipsite and minor chabazite and analcime), were used as starting materials. Mineralogical and chemical characterizations of IZ CLI and PHIL75 were reported elsewhere [41,52,53,54]. Modification of tuffs with CPyCl and ARQ resulted in surfactant modified natural zeolites. Used surfactant concentration was equivalent to 100% or 200% of zeolites’ ECEC value, necessary for monolayer and bilayer formation, respectively. Eight composites were prepared: IZ CLI-CPyCl-monolayer (CCM), IZ CLI-CPyCl-bilayer (CCB), IZ CLI-ARQ-monolayer (CAM), IZ CLI-ARQ-bilayer (CAB), PHIL75-CPyCl-monolayer (PCM), PHIL75-CPyCl-bilayer (PCB), PHIL75-ARQ-monolayer (PAM), and PHIL75-ARQ-bilayer (PAB) [41,42]. Starting materials and SMNZs were previously characterized by STA coupled with EGA, FTIR-ATR, and zeta potential measurements [41,42]. In this work, determination of the point of zero charge (pH_pzc_) of starting materials and SMNZs was performed. The pH_pzc_ was evaluated in accordance with the procedure given in the literature [55]. Experiments were performed by using KNO_3_ as an inert background electrolyte, at three concentrations: 0.001, 0.01, and 0.1 M [55]. For each concentration of KNO_3_, the initial pH (pH_i_) was adjusted using small amounts of 0.1 M HNO_3_/KOH. The measurements were performed on a pH/ion meter (781, Metrohm AG Metrohm AG, Herisau, Switzerland). Then, 10 Erlenmeyer flasks were used, each containing 50 mL of appropriate salt solution and 0.1 g of either starting materials or SMNZs. Suspensions were shaken at 300 rpm for 24 h at room temperature (shaker Unimax 1010, Heidolph Instruments, Schwabach, Germany), filtered using qualitative very slow filter paper (7970267, Lab Logistics Group GmbH, Meckenheim, Germany), and the final pH of each filtrate was measured (pH_f_). The pH_pzc_ of samples was determined as the pH value at the plateau of the curve pH_f_ = *f*(pH_i_).

Salicylic acid [CAS: 69-72-7] was of the analytical grade (≥98%), supplied from Merck Group (Darmstadt, Germany). The structural formula and basic physicochemical characteristics of SA are presented in Table 1.

The SA adsorption isotherm runs were carried out by addition of 25 mg of each SMNZ to 50 mL of SA solution—initial concentrations from 2 to 100 mg/L, under continuous shaking (300 rpm, room temperature) for 75 min. Samples were filtered and filtrates were kept for UV–VIS spectroscopy (Spectrophotometer UV—1800, Shimadzu, Kyoto, Japan), while absorbance was measured at λ = 296 nm.

To study the effect of temperature thermodynamic runs were conducted at—303.15, 313.15, 323.15, and 333.15K by mixing 25 mg of each adsorbent and 50 mL of SA solution (C_0_ = 20 mg/L), and suspensions were shaken for 75 min at 300 rpm in the incubator (Incubator 1000, Heidolph Instruments, Schwabach, Germany). Afterward, suspensions were filtered and filtrates analyzed by UV–VIS. Thermodynamic parameters: the change of standard free energy of adsorption (ΔG°), the change of standard enthalpy (ΔH°), and the change of standard entropy (ΔS°) were calculated. Kinetic runs of SA adsorption onto composites were evaluated as reported in our previous papers [41,42]. Briefly, 500 mg of each composite was mixed for 75 min with 1 L of drug water solution (C_0_ = 20 mg/L, pH~5). At fixed time intervals, 5 mL aliquots were taken, filtered, and tested on UV–VIS. Zeta potential of composites before and after SA adsorption (samples collected after kinetic runs) in water suspension (0.1 g/L) was evaluated using a Zetasizer Nano ZS90 (Malvern Panalytical, Malvern, UK). Additionally, pure SA and composites before and after SA adsorption were characterized by FTIR spectroscopy. All spectra were acquired using Nicolet iS50 spectrophotometer (Thermo Fisher Scientific, Waltham, United States) with a diamond attenuated total reflectance (ATR) accessory over a range 4000–400 cm^−1^ and with the resolution of 2 cm^−1^ and 64 scans. Omnic 9.2 software (Thermo Fisher Scientific, Waltham, MA, USA) was used to process spectra and the baseline and atmospheric influence (CO_2_ and H_2_O) correction was made.

## 3. Results and Discussion

### 3.1. Point of Zero Charge (pH_pzc_)

**Figure 1:**
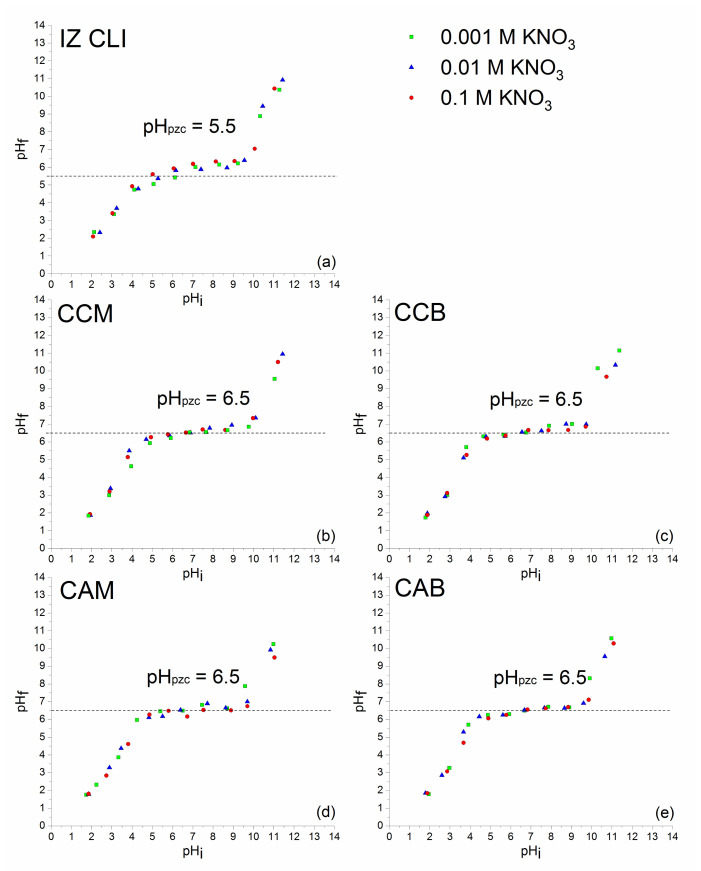
pH_f_ = *f*(pH_i_) plots for: (**a**) IZ CLI, (**b**) CCM, (**c**) CCB, (**d**) CAM, and (**e**) CAB. Experiments were carried out using three different concentrations of KNO_3_. pH_pzc_ values were additionally confirmed by plotting ΔpH_f_ = *f*(pH_i_) graphs (Supplementary Material, Figure S1).

**Figure 2:**
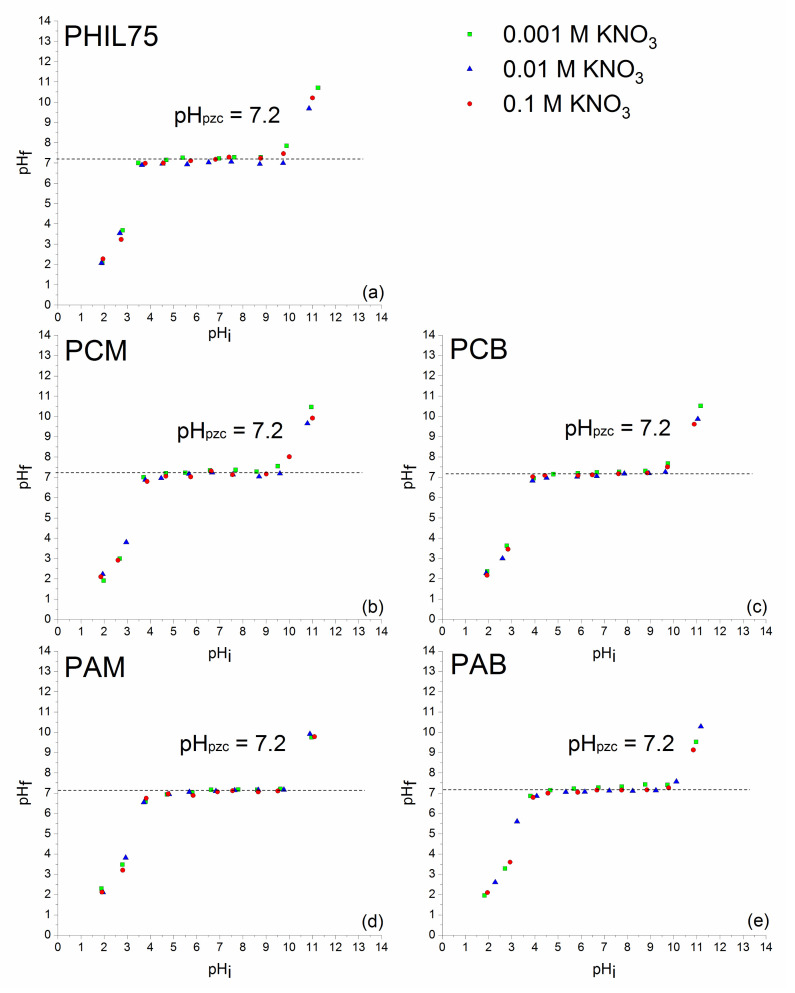
pH_f_ = *f*(pH_i_) plots for: (**a**) PHIL75, (**b**) PCM, (**c**) PCB, (**d**) PAM, and (**e**) PAB. Experiments were carried out using three different concentrations of KNO_3_. pH_pzc_ values were additionally confirmed by plotting ΔpH_f_ = *f*(pH_i_) graphs (Supplementary Material, Figure S2).

The removal efficacy of adsorbents for low polar molecules depends on the physicochemical properties of adsorbate molecules (polarity, solubility, dissociation constants, etc.). From Table 1, it can be seen that SA is an ionized compound, thus has different forms depending on pH values. Additionally, efficacy of adsorbents is dependent on their physical properties (the total charge and charge distribution, the size of pores, accessible surface area, etc.) [60,61,62]. The pH_pzc_ is also a significant property of minerals that defines the state of the surface of a dispersed solid phase at the solid-electrolyte solution surface [63]. The pH at which the sorbent surface has an equal amount of positive and negative active sites (the surface has zero net charge) is called pH_pzc_. In a certain range of solution pH, adsorbent will act as a buffer, trying to reach the pH_pzc_ value. For example, if solution pH is higher than pH_pzc_, the hydroxyl group will start to dissociate. The surface will become negatively charged; simultaneously, the concentration of protons in the solution will increase, leading to a decrease in pH value. If pH < pH_pzc_, hydroxyl groups will associate protons from the solution, the surface will become positive, while pH will increase to reach pH_pzc_ [64]. To comprehend if the modification of zeolite-rich tuffs (IZ CLI and PHIL75) with cationic surfactants had an influence on the surface charge of adsorbents, the pH_pzc_ values were determined for the starting zeolite-rich tuffs as well as the composites and presented in Figure 1 and Figure 2.

From Figure 1 and Figure 2, it can be seen that for all adsorbents, curves pH_f_ = *f*(pH_i_) show the same shape and have a plateau that represents the pH range where the zeolitic surface will behave as a buffer. The increase of the final pH (pH_f_) with the increase of the initial pH (pH_i_) up to ~5.0 was observed for the starting clinoptilolite and its modified samples, where the plateau began, except for CAB, where the plateau starts at ~4.5. For all clinoptilolite samples, plateaus end at pH around 10.0. Within these values, pH_f_ were independent on the pH_i_. For starting phillipsite and its modified zeolites, plateaus were observed from ~3.5 to ~9.8. At plateaus, all adsorbents exhibit amphoteric properties. The plateaus of the curves correspond to the pH range where the buffering effect of the zeolitic surface takes place—i.e., where for all pH_i_ in this range, pH_f_ is almost the same and corresponds to pH_pzc_ [64]. By comparing the pH_pzc_ values of the starting clinoptilolite (pH_pzc_ = 5.5) and its four modified zeolites (pH_pzc_ = 6.5), and the starting phillipsite (pH_pzc_ = 7.2) and its composites (pH_pzc_ = 7.2), it can be seen that cationic surfactants present at the clinoptilolite surfaces slightly increase the pH_pzc_ of the zeolitic tuff. For phillipsite samples, pH_pzc_ was not changed after adsorption of surfactants ions. According to Daković et al. (2010), these results suggest that SMNZs have similar surface functional groups with similar acid and basic characteristics as starting materials [64]. Since SA adsorption by SMNZs was followed in distilled water (pH around 5), the results of the pH_pzc_ suggest that the surfaces of SMNZs are uncharged at this pH value. Additionally, different ionic strengths of electrolyte KNO_3_ were not influencing the pH_f_ = *f*(pH_i_) curves; in other words, the curves were overlapping and this is an indication that K^+^ or NO_3_^−^ ions were not specifically adsorbed on SMNZs surfaces [63,65].

The obtained results for pH_pzc_ of starting zeolites and their SMNZs were in agreement with the literature data. For example, modification of clinoptilolite with HDTMA (0.25 L of a 0.03 M HDTMA solution was mixed with 25 g of zeolite tuff) did not change pH_pzc_—it remained around 6.9 [66]. Clinoptilolite modified with benzalkonium chloride (below and equal to ECEC of clinoptilolite) had the same pH_pzc_ values of 6.8 for all composites [67]. In another study, clinoptilolite was modified with three levels of octadecyldimethylbenzyl ammonium (ODMBA) (below and equal to ECEC value of clinoptilolite), and pH_pzc_ values changed from 6.8 ± 0.1 for starting clinoptilolite to 7.0 ± 0.1 for the three organozeolites [64].

### 3.2. Drug Adsorption

#### 3.2.1. Adsorption Isotherms of SA on Composites

**Figure 3:**
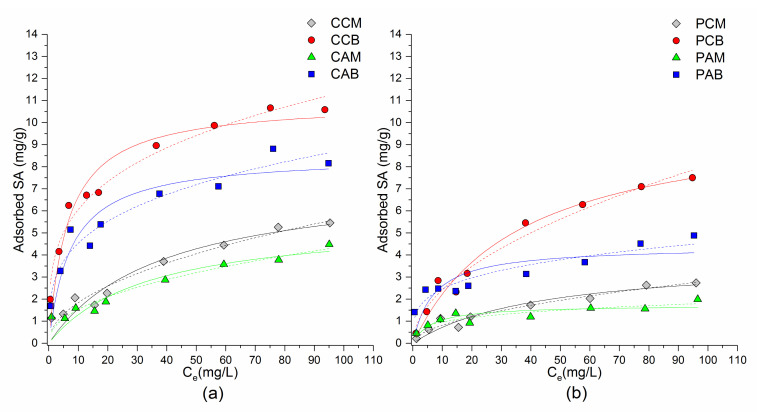
Equilibrium adsorption of SA on: (**a**) SMNZs of IZ CLI; (**b**) SMNZs of PHIL75. Solid line = Langmuir model; dotted line = Freundlich model.

Adsorption isotherms of SA from water solution by IZ CLI and PHIL75 composites are presented in Figure 3. Results suggested that for all samples, adsorption of SA increased when the initial drug concentration increased. Additionally, adsorption of SA increased with increasing of the amount of both surfactants in SMNZs. It was noticed that adsorption of SA was higher for SMNZs obtained from IZ CLI than for PHIL75 modified zeolites, probably due to the higher zeolitic (clinoptilolite) content in IZ CLI. Additionally, drug adsorption followed orders: CCB > CAB > CCM > CAM and PCB > PAB > PCM > PAM, making it easier to notice that bilayer composites had higher adsorption capacity than monolayer composites, showing that the amount of used surfactant for modification of the tuffs is a very important parameter. Another observation is that composites with CPyCl had better adsorption compared to the ones with ARQ, this can be explained by different chemical structures of used surfactants, which are influencing their different arrangements at the zeolitic surface [42], consequently leading to different adsorption affinity for SA. The highest adsorption was observed for IZ CLI and PHIL75 containing CPyCl in amount equal to 200% of zeolites ECEC value (samples CCB and PCB). On these adsorbents, maximum adsorbed amounts of SA were 10.6 and 7.5 mg/g, for CCB and PCB, respectively. Results confirmed the fact that adsorption of relatively nonpolar molecules is influenced by the physical properties of zeolites and the type of used surfactant [34,41,63,68,69].

Adsorption of SA followed nonlinear isotherms, for this reason Langmuir and Freundlich isotherm models were used to fit the experimental data and are given with the equations

(1) $$Q_{e}=\frac{Q_{max}K_{L}C_{e}}{1+K_{L}C_{e}}$$

(2) $$Q_{e}=K_{F}C_{e}{}^{\frac{1}{n}}$$

where $Q_{e}$ (mg/g) is the amount of loaded adsorbate, $C_{e}$ mg/L) is the concentration of adsorbate in solution at equilibrium, *Q_max_* (mg/g) is the highest concentration of adsorbate, *K_L_* (L/mg) and *K_F_* (L/mg) are Langmuir and Freundlich constants, respectively. While 1/*n* is a constant associated with adsorption intensity [70].

Langmuir isotherm (Equation (1)) is based on assumption that adsorbate molecules can form only one layer, and for this reason, the curve reaches a plateau [41,42]. Freundlich isotherm (Equation (2)) takes into consideration the heterogeneity of adsorbents. The constant n in the Freundlich equation is a constant that represents the parameter characterizing quasi-Gaussian energetic heterogeneity of the adsorption surface [71]. The amount of adsorbed molecule is the summation of adsorption on all sites (different bonding energies), with the stronger binding sites being occupied first, until adsorption energy exponentially decreases upon the completion of the adsorption process. The n constant gives an indication of the favorability of adsorption and it is stated that n in the range of 2–10 represent good, 1–2 moderately difficult, and less than 1 poor adsorption characteristics [71]. Chosen mathematical models were evaluated considering the determination coefficients (R^2^), and Akaike weight parameter (AIC*w*—higher the value, a mathematical model is considered as a better approximation of experimental data) [72,73]. The calculated parameters of adsorption isotherms are given in Table 2, and based on AIC*w* values, in most cases a better fit was achieved with the Freundlich model. On the other hand, although slightly lower correlation coefficients were achieved with Langmuir model, a good agreement of the experimental data was also obtained with this model.

Nonlinear isotherms were also reported for adsorption of SA on different organomodified minerals, and an overview on literature, together with data from this study, is given in Table 3. According to literature, nonlinear isotherms obtained for adsorption of relatively nonpolar molecules (such as NSAIDs) onto the SMNZs indicate a complex adsorption mechanism that includes hydrophobic partitioning and ion exchange [63,68,69,74]. SA is a relatively nonpolar molecule and exists in the anionic form at pH > 3 (see Table 1). Thus, nonlinear isotherms for SA (Figure 3) indicate that in the case of monolayer composites, SA is adsorbed through electrostatic interactions with the surfactant cationic heads attached directly to the zeolite surface and also through hydrophobic interactions between the surfactant chains and hydrophobic parts of SA. Simultaneously with this process, additional interaction happens, especially when bilayer composites are used as adsorbents—counter anions in surfactant molecules (such as chlorides) are exchanged with the anionic form of the investigated drug. The higher SA adsorption capacities for bilayer composites confirmed that anion exchange contributes significantly to the overall adsorption process.

#### 3.2.2. Thermodynamic Runs

The data collected for adsorption of SA on composites at four temperatures (303.15, 313.15, 323.15, and 333.15 K) were used to estimate thermodynamic parameters. The changes of standard free energy of adsorption (ΔG°), standard enthalpy (ΔH°), and standard entropy (ΔS°) were calculated from the equations

ΔG° = −R*T*ln*K* (3)

ln*K* = ΔS°/R − ΔH°/(RT) (4)

where *T* is the temperature in Kelvin (*K*), R is the gas constant (8.314 J/mol K), and *K* is the partitioning coefficient (the ratio of the equilibrium amount of adsorbed drug and equilibrium concentration of drug remaining in solution). ΔG° was calculated from Equation (3), whereas ΔS° and ΔH° were estimated from the slope and intercept of the plots $\ln K=f\left(\frac{1}{T}\right)$ Equation (4)) [77]. The values of thermodynamic parameters for the adsorption of SA are given in Table 4.

All composites had the positive values of ΔG° indicating that the adsorption of SA is a nonspontaneous process [78]. It is observed that for monolayer composites ΔG° values were higher than for bilayer samples, suggesting that for CCM, CAM, PCM, and PAM adsorption of SA is an even less spontaneous process. Additionally, the change of standard free energy increased as temperature increased, leading to the conclusion that adsorption is less favorable at higher temperatures. Considering ΔH°, almost all composites had negative values indicating that SA adsorption is exothermic [79]. Calculated positive ΔH° values were obtained only for CAB and PAM samples. The ΔS° values were negative for all adsorbents, suggesting the decrease of randomness at the solid/liquid interface during the adsorption of SA onto composites, which is probably caused by more regular arrangement of SA molecules onto their surfaces [79,80].

#### 3.2.3. Kinetic Runs

**Figure 4:**
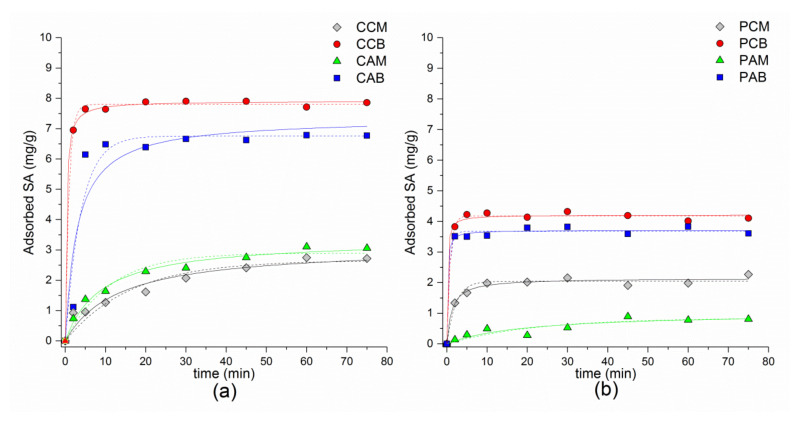
Kinetics run for SA adsorption on: (**a**) composites of IZ CLI; (**b**) composites of PHIL75. Solid line = pseudo-second order; dotted line = pseudo-first order.

The effect of contact time on SA adsorption onto SMNZs is shown in Figure 4. Results indicate that when the amount of cationic surfactant used in the modification of starting materials was 200% of their ECEC values (CCB, CAB, PCB, and PAB), the adsorption rate increased rapidly and adsorption equilibrium was reached in approximately 10–15 min.

Adsorption of SA was also very fast for PHIL75 monolayer sample (PCM), while for PAM, equilibrium was reached after 45 min, whereas for IZ CLI monolayer composites (CCM and CAM) more time was necessary (~60 min). For the initial SA concentration of 20 mg/L (40 mg/g), higher adsorption of SA was observed for IZ CLI composites than for PHIL75 modified samples. Among IZ CLI composites, maximum adsorbed amounts of drug were 7.8 and 6.8 mg/g for CCB and CAB, respectively. Monolayer samples showed maximum adsorbed amounts of SA—3.1 mg/g (CAM) and 2.7 mg/g (CCM). Similar behavior was noticed when PHIL75 was used as starting material. Bilayer composite with CPyCl (PCB), showed maximum adsorbed amount of SA of 4.2 mg/g, while composite containing ARQ bilayer (PAB) adsorbed slightly less amount of drug (3.6 mg/g). Between monolayer samples, composite with CPyCl (PCM) adsorbed 2.2 mg/g of SA, while for PAM sample adsorption of SA was 0.8 mg/g.

The kinetic runs for SMNZs are often described with a pseudo-first order (PFO) and a pseudo-second order (PSO) model [36,38,81,82,83], given by the equations

(5) $$Q_{t}=Q_{max}\left(1-e^{-K_{1}t}\right).$$

(6) $$Q_{t}=\frac{K_{2}Q_{max}^{2}t}{1+K_{2}Q_{max}t}.$$

where *Q_t_* is the amount of drug adsorbed by adsorbent (mg/g) as a function of time *t* (min), *Q_max_* is adsorbate concentration at equilibrium, whereas *K*_1_ and *K*_2_ are the pseudo-first and the pseudo-second-order constants, respectively.

Based on statistical criteria (Table 5), better goodness-of-fit was achieved by PSO. Good agreement with PSO model was also reported in our previously published results on adsorption of DCF, IBU, KET, and NAP by the same composites [41,42]. Pseudo-second order model was also successful in describing kinetics when IBU was adsorbed on phillipsite-rich tuff [39] or clinoptilolite-rich tuff [84] modified with CPyCl, benzalkonium chloride, or HDTMA-Cl/Br.

### 3.3. Characterization after Adsorption

#### 3.3.1. Zeta Potential

**Figure 5:**
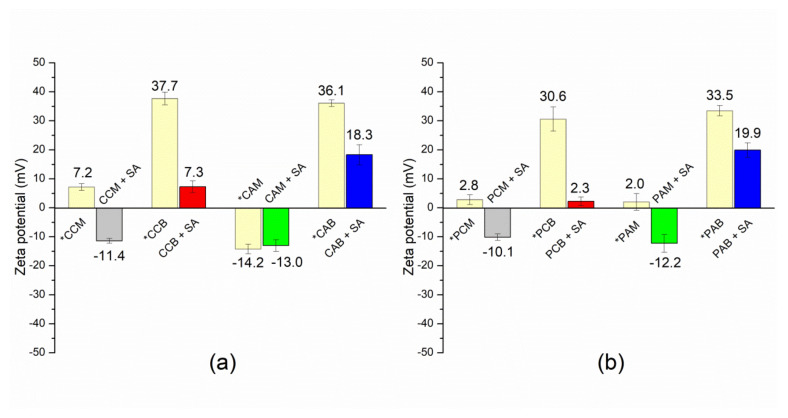
Zeta potential measured for: (**a**) IZ CLI composites, and (**b**) PHIL75 composites after adsorption of SA. Measured values were compared with zeta potential of the composites (before drug adsorption (*) previously reported in Smiljanić et al. (2020) [42]. Data were used with permission from Microporous and Mesoporous Materials, Elsevier, license number: 5207040046204.

Zeta potential values for composites before and after adsorption of SA are illustrated in Figure 5.

Results suggest that adsorption of SA caused changes in the surface charge of all surfactant modified zeolites, providing evidence that SA molecules are present at the surface of composites. It is observed, that for bilayer composites containing CPyCl—after adsorption of SA—zeta potential changed from +37.7 mV for sample CCB to +7.3 mV, while for PCB, zeta potential changed from +30.6 mV to +2.3 mV. For monolayer composite CCM, zeta potential (+7.2 mV) becomes negative after adsorption of SA (−11.4 mV), while for PCM, zeta potential changed from +2.8 mV to −10.1 mV after adsorption of SA. When SA was adsorbed on ARQ bilayer composites, zeta potential changed from +36.1 mV for sample CAB to +18.3 mV, while for PAB, from +33.5 mV to +19.9 mV. Adsorption of the drug on composites containing ARQ (amount equivalent to 100% of ECEC value) changed potential from −14.2 mV to −13.0 mV (sample CAM) and from +2.0 mV to −12.2 mV (sample PAM). Differences in values of zeta potential after adsorption of SA on IZ CLI and PHIL75 composites may be caused by the different arrangement of and ARQ ions at their surfaces and are the consequence of the different structures of surfactants (CPyCl contains one alkyl chain, while ARQ possesses two alkyl chains). Thus, when the amount of CPyCl was equal to ECEC value (CCM and PCM), zeta potential was close to zero confirming nearly monolayer formation and almost complete hydrophobicity of both zeolitic surfaces [35]. When ARQ amount was 100% ECEC, potential of clinoptilolite sample (CAM) was negative, while for phillipsite sample (PAM) became close to zero. This may be an indication that ARQ ions are adsorbed as micelle at clinoptilolite surface, leaving part of the surface uncovered. For phillipsite, which possess more available sites for ARQ adsorption, surfactant ions are uniformly adsorbed forming a monolayer. When the amount of both surfactants was 200% of the ECEC value, zeta potential became positive, pointing to charge reversal and bilayer formation at clinoptilolite and phillipsite surfaces. After adsorption of SA, zeta potential of samples containing monolayer (CCM, PCM, and PAM) or micelle (CAM), became or stayed negative; while for all bilayer composites, zeta potential decreased but was still positive. The decrease of zeta potential for bilayer composites after adsorption of SA was more pronounced for composites containing CPyCl (CCB and PCB) indicating that hydrophobic part of SA and its anionic form are involved in adsorption. For ARQ bilayer composites (CAB and PAB), hydrophobic interactions of the hydrophobic part of SA with alkyl chains dominate, while the anionic form of SA contributes less to the overall adsorption.

#### 3.3.2. FTIR-ATR

**Figure 6:**
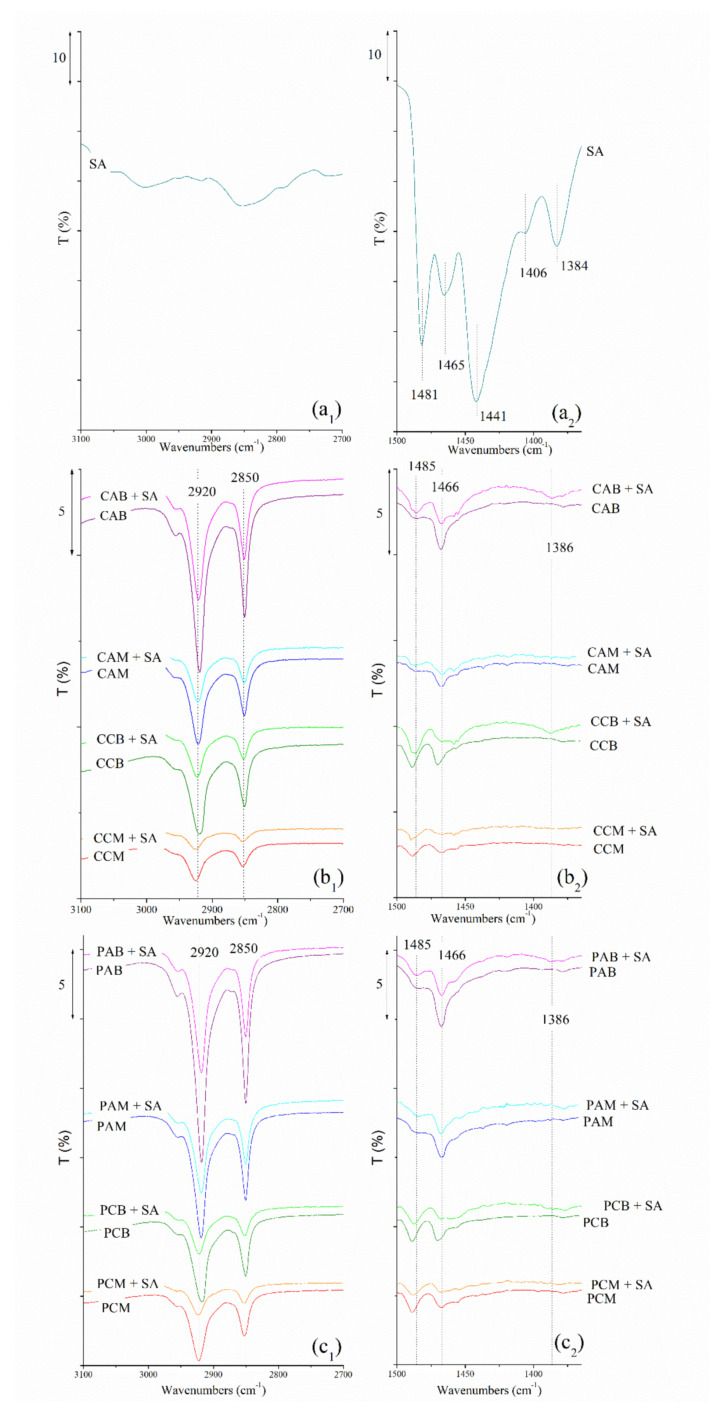
FTIR–ATR spectra of: SA (*a_1_ and a_2_), and spectra of IZ CLI composites before and after adsorption of SA (*b_1_ and b_2_), and spectra of PHIL75 composites before and after adsorption of SA (*c_1_ and c_2_). *Subscript 1 relates to the spectral range 3100–2700 cm^−1^ and subscript 2 is connected to the range 1500–1360 cm^−1^ of the same spectra.

FTIR-ATR spectra for SA, and composites before and after adsorption of SA, are given in Figure 6. The results have shown that after adsorption, peaks at 2920 cm^−1^ and 2850 cm^−1^ (symmetric and asymmetric C-H stretching vibrations), and at 1466 cm^−1^ (C-H bending vibration) originating from surfactant present at the zeolitic surface [42] were reduced. Most likely this happened because small amounts of the loosely bound surfactant molecules were released during the adsorption experiment [37]. Additionally, small changes in spectra after adsorption, in the region between 1490 cm^−1^ and 1380 cm^−1^, evidenced the adsorption of SA. For example, a low-intensity peak at 1386 cm^−1^ characteristic for this drug (slightly shifted from 1384 cm^−1^) has appeared in bilayer composites: CCB + SA, CAB + SA, and PAB + SA, while in the case of monolayer composites this peak was not visible probably due to the lower adsorption of SA onto these composites. Additionally, incorporation of SA in composites CAB and PAB slightly increased the intensity of peak at 1486 cm^−1^ (C-H bending vibration). No relevant variations in wavenumbers of the bands assigned to zeolite-rich tuffs after drug incorporation were observed, indicating that zeolitic framework was unaltered.

## 4. Conclusions

In this study, previously prepared and characterized monolayer and bilayer composites of natural zeolites and cationic surfactants, CPyCl and ARQ, were tested for the removal of SA—an emerging contaminant. Additional characterization—namely determination of pH_pzc_—was performed and the results have suggested that experimental conditions, such as pH, could play a significant role in the adsorption process. Based on adsorption isotherm, the highest adsorption of SA was achieved by the bilayer composites suggesting hydrophobic partitioning and ion exchange as two simultaneous adsorption mechanisms. Adsorption capacities of tested SMNZs were in a range from 1.7 to 11 mg/g. Kinetic runs have suggested that adsorption of SA is a fast process and a good correlation of experimental data with pseudo-second order was confirmed, while thermodynamic parameters suggest that adsorption of SA onto composites is a nonspontaneous, exothermic process followed by a decrease in randomness at the solid/liquid interface. Characterization of SMNZs after adsorption of SA confirmed drug adsorption and that there were no structural changes in zeolitic framework. Results showed that SMNZs have an affinity for the removal of SA and potential for decontamination of polluted water.

## Figures and Tables

**Table 1:** Structural formula and physicochemical properties of salicylic acid

Structure	M (g/mol)	^4^ Solubility (g/L)	^1,2^ pKa	^2,3^ log K_ow_
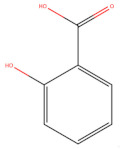	138.12	2.24	2.93	2.26

^1^ [56], ^2^ [57], ^3^ [58], ^4^ [59].

**Table 2:** Mathematical models for the adsorption isotherms: parameters and goodness-of-fit

Drug	Sample	Model	Parameters			Goodness-of-Fit	
			*K* (L/mg)	*n*	*Q_max_* (mg/g)	R^2^	AIC*w*
**SA**	CCM	Langmuir	0.03 ± 0.01		8 ± 2	0.905	0.033
		Freundlich	0.6 ± 0.2	2.0 ± 0.3		0.955	0.967
	CCB	Langmuir	0.15 ± 0.04		11.0 ± 0.6	0.936	0.030
		Freundlich	3.2 ± 0.3	3.7 ± 0.3		0.970	0.970
	CAM	Langmuir	0.03 ± 0.01		5.6 ± 0.9	0.851	0.029
		Freundlich	0.5 ± 0.2	2.2 ± 0.3		0.932	0.971
	CAB	Langmuir	0.14 ± 0.05		8.5 ± 0.7	0.865	0.034
		Freundlich	2.3 ± 0.3	3.5 ± 0.4		0.936	0.966
	PCM	Langmuir	0.023 ± 0.009		3.8 ± 0.6	0.926	0.095
		Freundlich	0.24 ± 0.06	1.9 ± 0.2		0.955	0.905
	PCB	Langmuir	0.028 ± 0.006		10.0 ± 1	0.970	0.541
		Freundlich	0.7 ± 0.2	1.9 ± 0.2		0.969	0.459
	PAM	Langmuir	0.16 ± 0.08		1.7 ± 0.2	0.713	0.100
		Freundlich	0.5 ± 0.1	3.6 ± 0.7		0.824	0.900
	PAB	Langmuir	0.14 ± 0.08		4.4 ± 0.6	0.579	0.002
		Freundlich	1.3 ± 0.2	3.8 ± 0.6		0.891	0.998

**Table 3:** Bibliography data on SA adsorption onto surfactant modified minerals

Starting Mineral	Surfactant	*Q_max_* of Prepared Organominerals	Reference
**Montmorillonite-KSF**	HDTMA	42.4 mmol/kg (5.9 mg/g)	[51]
**Commercial zeolite**	HDTMA	23.8 mmol/kg (3.3 mg/g)	[51]
**Bentonite**	ODMBA	2.3·× 10^−5^ mol/g (3.2 mg/g)	[75]
**Kaolin**	ODMBA	3.5·× 10^−5^ mol/g (4.8 mg/g)	[75]
**Synthetic zeolite Y**	Modified with metal cations (sodium, cobalt, nickel, or copper) and subsequently with CPyCl	1.2–3.0 mg/g	[76]
**Clinoptilolite**	Benzalkonium chloride	13 and 19 mg/g	[50]
**Clinoptilolite-rich tuff**	CPyCl or ARQ	5.6–11 mg/g	Current study
**Phillipsite-rich tuff**	CPyCl or ARQ	1.7–10 mg/g	Current study

**Table 4:** Thermodynamic parameters

Adsorbent	*T* (K)	ΔG° (kJ/mol)	ΔH° (kJ/mol)	ΔS° (J/(mol·K))
CCM	303.15	8.1 ± 0.4	−17 ± 12	−84 ± 36
	313.15	9 ± 2		
	323.15	9 ± 1		
	333.15	10.7 ± 0.9		
CCB	303.15	3.8 ± 0.2	−15 ± 2	−64 ± 5
	313.15	4.8 ± 0.3		
	323.15	5.18 ± 0.09		
	333.15	5.7 ± 0.3		
CAM	303.15	8.2 ± 0.5	−14.0 ± 0.8	−73 ± 3
	313.15	9 ± 2		
	323.15	10 ± 2		
	333.15	10 ± 2		
CAB	303.15	4.7 ± 0.3	2 ± 3	−8 ± 8
	313.15	4.9 ± 0.2		
	323.15	4.84 ± 0.04		
	333.15	5.2 ± 0.3		
PCM	303.15	8.72 ± 0.09	−34.4 ± 0.3	−142 ± 2
	313.15	10 ± 2		
	323.15	12 ± 2		
	333.15	13.0 ± 0.2		
PCB	303.15	6.1 ± 0.3	−22 ± 3	−92 ± 8
	313.15	6.9 ± 0.4		
	323.15	7.9 ± 0.5		
	333.15	9 ± 1		
PAM	303.15	13 ± 2	0.67 ± 15	−37 ± 45
	313.15	10.9 ± 0.5		
	323.15	13 ± 2		
	333.15	13 ± 2		
PAB	303.15	6.1 ± 0.9	−2 ± 5	−27 ± 15
	313.15	8 ± 3		
	323.15	7.2 ± 0.6		
	333.15	7.4 ± 0.2		

**Table 5:** Mathematical models of kinetic runs: parameters and goodness-of-fit

Drug	Sample	Model	Parameters			Goodness-of-Fit		* RE (%)
			*K*_1_ (min ^−1^)	*K*_2_ (g mg ^−1^ min ^−1^)	*Q_max_* (mg/g)	R^2^	AIC*w*	
**SA**	CCM	PFO	0.06 ± 0.02		2.7 ± 0.2	0.904	0.153	6.63
		PSO		0.02 ± 0.01	3.1 ± 0.3	0.934	0.847	
	CCB	PFO	1.10 ± 0.07		7.80 ± 0.05	0.998	0.246	18.35
		PSO		0.49 ± 0.07	7.91 ± 0.05	0.999	0.754	
	CAM	PFO	0.09 ± 0.02		2.9 ± 0.2	0.955	0.011	7.30
		PSO		0.033 ± 0.007	3.4 ± 0.2	0.983	0.989	
	CAB	PFO	0.26 ± 0.07		6.8 ± 0.4	0.913	0.870	16.49
		PSO		0.05 ± 0.03	7.4 ± 0.6	0.947	0.130	
	PCM	PFO	0.46 ± 0.08		2.05 ± 0.06	0.962	0.151	5.47
		PSO		0.38 ± 0.08	2.14 ± 0.06	0.974	0.849	
	PCB	PFO	1.2 ± 0.2		4.18 ± 0.04	0.995	0.889	10.03
		PSO		1.6 ± 0.8	4.21 ± 0.06	0.992	0.111	
	PAM	PFO	0.05 ± 0.03		0.8 ± 0.2	0.797	0.406	1.97
		PSO		0.06 ± 0.05	1.1 ± 0.3	0.813	0.594	
	PAB	PFO	1.6 ± 0.5		3.67 ± 0.05	0.988	0.257	8.73
		PSO		1.9 ± 0.9	3.71 ± 0.06	0.991	0.743	

* RE—removal efficiency, calculated as $\mathrm{RE}\;\left(\%\right)=\frac{C_{o}-C_{}}{C_{o}}*100$ where *C_o_* is the initial concentration of drug solution, and *C* is the last measured concentration.

## Data Availability

The data presented in this study are available on the request from the corresponding author.

## Supplementary Materials

The following are available online at https://www.mdpi.com/article/10.3390/ma14247728/s1, Figure S1. ΔpHf = *f*(pHi) plots for: (a) starting material IZ CLI, (b) CCM, (c) CCB, (d) CAM, and (e) CAB). Experiments were carried out using three different concentrations of KNO3 (0.001 M, 0.01 M, and 0.1 M). pHpzc value of each material was taken as the average value of the curves` intercept with the x-axis; Figure S2. ΔpHf = *f*(pHi) plots for: (a) starting material PHIL 75, (b) PCM, (c) PCB, (d) PAM, and (e) PAB). Experiments were carried out using three different concentrations of KNO_3_ (0.001 M, 0.01 M, and 0.1 M). pH_pzc_ value of each material was taken as the average value of the curves` intercept with the x-axis.
