# Supplementary material for: Application of Surfactant Modified Natural Zeolites for the Removal of Salicylic Acid—A Contaminant of Emerging Concern
Source: Materials (Basel). 2021 Dec 14;14(24):7728. doi: 10.3390/ma14247728 (PMC8708488; doi:10.3390/ma14247728)
Supplement: Supplementary file 1 [file materials-14-07728-s001.zip › materials-1463796-supplementary.pdf]

materials

Supplementary Materials

pplication of Surfactant Modified Natural Zeolites for the Removal of Salicylic Acid—A Contaminant of Emerging Concern

Danijela Smiljanić [1,*], Aleksandra Daković [1], Milena Obradović [1], Milica Ožegović [1], Francesco Izzo [2], Chiara Germinario [3], and Bruno de Gennaro [4]

[1] Institute for Technology of Nuclear and Other Mineral Raw Materials, Franche D' Epere 86, 11000 Belgrade, Serbia; a.dakovic@itnms.ac.rs (A.D.); m.obradovic@itnms.ac.rs (M.O.); m.spasojevic@itnms.ac.rs (M.O.)
[2] Department of Earth Sciences, Environment and Resources, Federico II University, Via Cinthia, 80126 Napoli, Italy; francesco.izzo4@unina.it
[3] Department of Science and Technology, University of Sannio, Via F. De Sanctis, 82100 Benevento, Italy; chiara.germinario@unisannio.it
[4] Department of Chemical, Materials and Production Engineering, Federico II University, Piazzale V. Tecchio 80, 80125 Naples, Italy; bruno.degennaro@unina.it
* Correspondence: d.smiljanic@itnms.ac.rs

Citation: Smiljanić, D.; Daković, A.; Obradović, M.; Ožegović, M.; Izzo, F.; Germinario, C.; de Gennaro, B. Application of Surfactant Modified Natural Zeolites for the Removal of Salicylic Acid—A Contaminant of Emerging Concern. *Materials* **2021**, *14*, 7728. https://doi.org/10.3390/ma14247728

Academic Editor: Lucjan Chmielarz

Received: 29 October 2021
Accepted: 10 December 2021
Published: 14 December 2021

Publisher's Note: MDPI stays neutral with regard to jurisdictional claims in published maps and institutional affiliations.

Copyright: © 2021 by the authors. Licensee MDPI, Basel, Switzerland. This article is an open access article distributed under the terms and conditions of the Creative Commons Attribution (CC BY) license (http://creativecommons.org/licenses/by/4.0/).

The Addition to the Section:

3.1. The Point of Zero Charge (pH_{pzc})

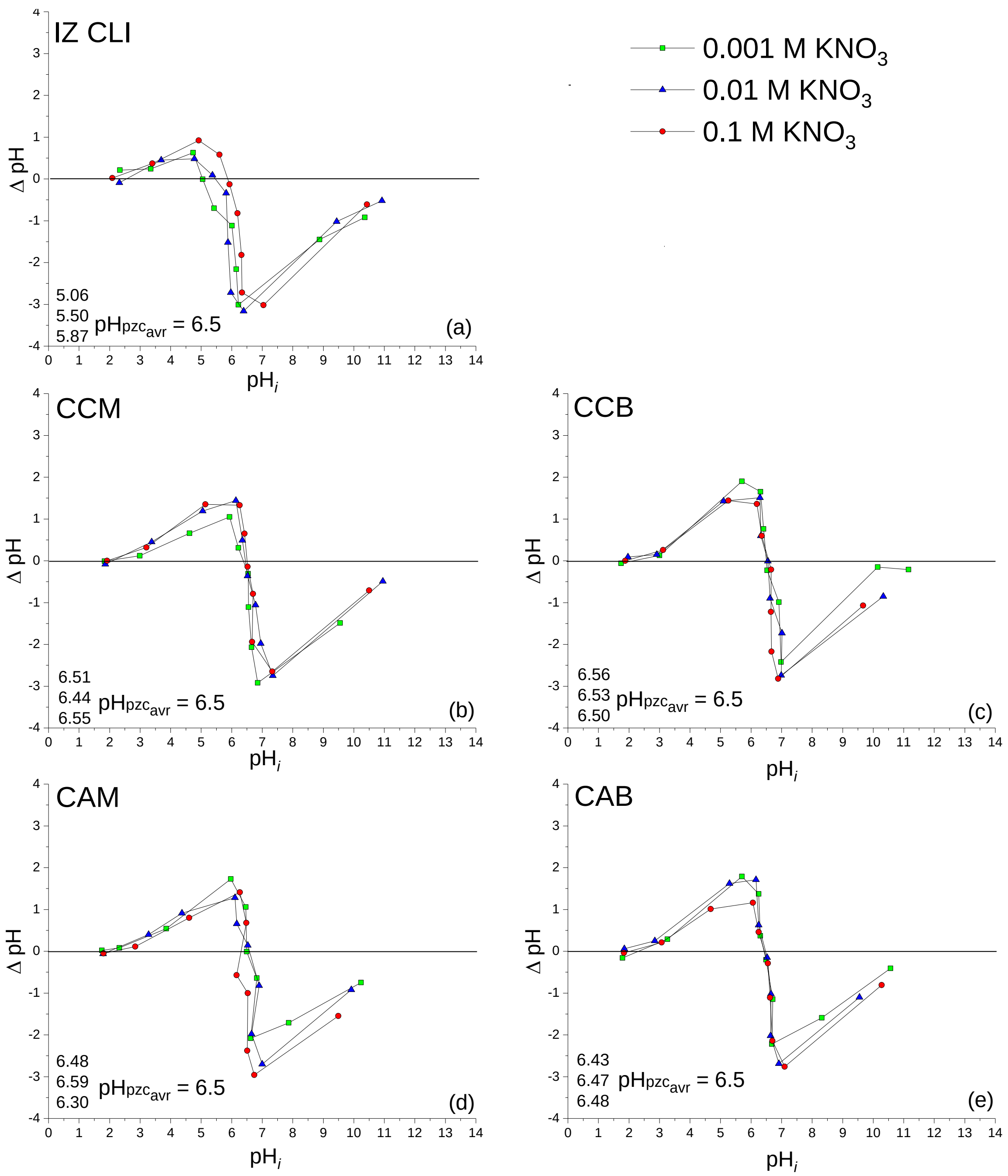

Figure S1. $\Delta pH_f = f(pH_i)$ plots for: (**a**) starting material IZ CLI, (**b**) CCM, (**c**) CCB, (**d**) CAM, and (**e**) CAB). Experiments were carried out using three different concentrations of KNO_3 (0.001 M, 0.01 M, and 0.1 M). pH_{pzc} value of each material was taken as the average value of the curves` intercept with the x-axis.

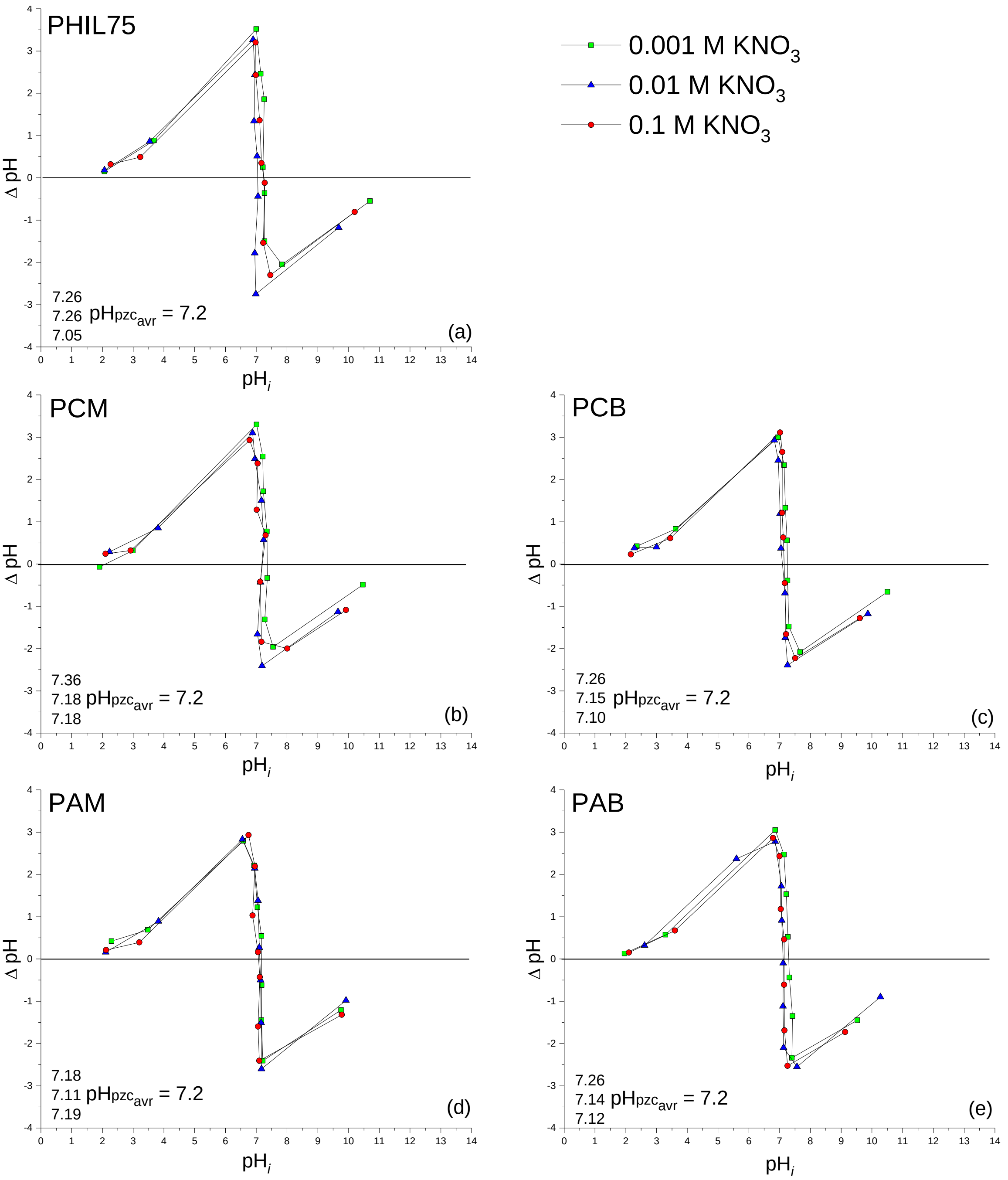

Figure S2. $\Delta pH_f = f(pH_i)$ plots for: (**a**) starting material PHIL 75, (**b**) PCM, (**c**) PCB, (**d**) PAM, and (**e**) PAB). Experiments were carried out using three different concentrations of KNO_3 (0.001 M, 0.01 M, and 0.1 M). pH_{pzc} value of each material was taken as the average value of the curves` intercept with the x-axis.
